# Supplementary material for: Splice-Junction-Based Mapping of Alternative Isoforms in the Human Proteome
Source: Cell Rep. Author manuscript; Available in PMC 2020 Jan 15. (PMC6961840; doi:10.1016/j.celrep.2019.11.026)
Supplement: 2 [file NIHMS1546469-supplement-2.zip › DF1/PXD010154/Esophagus/Esophagus_4_PALLD_FKEDLLNNGQPIYWFK.pdf]

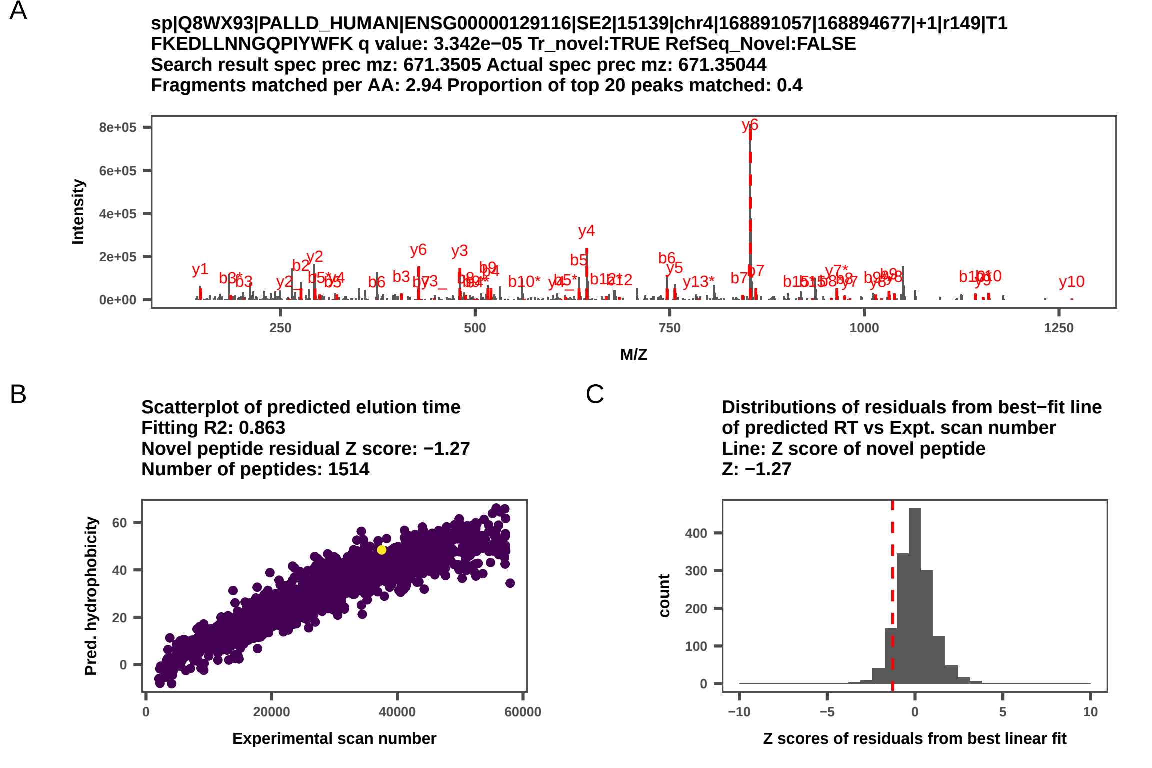
A
sp|Q8WX93|PALLD_HUMAN|ENSG00000129116|SE2|15139|chr4|168891057|168894677|+1|r149|T1
FKEDLLNNGQPIYWFK q value: 3.342e−05 Tr_novel:TRUE RefSeq_Novel:FALSE
Search result spec prec mz: 671.3505 Actual spec prec mz: 671.35044
Fragments matched per AA: 2.94 Proportion of top 20 peaks matched: 0.4
Intensity
8e+05
6e+05
4e+05
2e+05
0e+00
250
500
750
1000
1250
M/Z
B
Scatterplot of predicted elution time
Fitting R2: 0.863
Novel peptide residual Z score: −1.27
Number of peptides: 1514
Pred. hydrophobicity
60
40
20
0
20000
40000
60000
Experimental scan number
C
Distributions of residuals from best−fit line
of predicted RT vs Expt. scan number
Line: Z score of novel peptide
Z: −1.27
count
400
300
200
100
0
−10
−5
0
5
10
Z scores of residuals from best linear fit
